# Analytical characterization and reference interval of an enzyme-linked immunosorbent assay for active von Willebrand factor

**DOI:** 10.1371/journal.pone.0211961

**Published:** 2019-02-13

**Authors:** Lisa N. van der Vorm, Li Li, Dana Huskens, Walid Chayouâ, Hilde Kelchtermans, Philip G. de Groot, Mark Roest, Jasper A. Remijn, Bas de Laat

**Affiliations:** 1 Cardiovascular Research Institute Maastricht, Maastricht University Medical Centre, Maastricht, The Netherlands; 2 Synapse Research Institute, Maastricht, The Netherlands; 3 Department of Clinical Chemistry and Hematology, Gelre Hospitals, Apeldoorn, The Netherlands; 4 Department of Clinical Chemistry, Meander Medical Center, Amersfoort, The Netherlands; Institut d'Investigacions Biomediques de Barcelona, SPAIN

## Abstract

**Background:**

Interaction of von Willebrand factor (VWF) with platelets requires a conformational change that exposes an epitope within the VWF A1 domain, enabling platelet glycoprotein Ibα binding. Quantification of this ‘‘active” conformation of VWF has been shown to provide pathophysiological insight into conditions characterized by excessive VWF-platelet interaction.

**Methods:**

We developed an immunosorbent assay based on a variable heavy chain antibody fragment against the VWF A1 domain as a capture antibody. Assay performance in terms of specificity (binding to active R1306W- and sheared VWF), precision, accuracy, linearity, limits of detection and stability were determined. Active VWF, VWF antigen, VWF ristocetin cofactor activity, VWF:GP1bM and VWF propeptide were measured in citrated plasma and platelet-VWF binding in whole blood from 120 healthy individuals to establish a reference interval for active VWF and to assess associations with other VWF parameters.

**Results:**

Intra- and inter-assay CVs were between 2.4–7.2% and 4.1–9.4%, depending on the level. Mean recovery of spiked recombinant R1306W VWF was 103±3%. The assay was linear in the range of 90.1–424.5% and had a limit of quantification of 101%. The reference interval for active VWF was 91.6–154.8% of NPP. Significant, positive correlations between active VWF and all other VWF parameters were found, with the strongest correlation with VWF:GP1bM binding.

**Conclusions:**

We developed and validated an immunosorbent assay for the accurate detection of active VWF levels in plasma. The assay fulfilled all analytical criteria in this study and a reference interval was established, allowing its use to quantify active VWF in pathological conditions for future research.

## Introduction

Von Willebrand factor (VWF) is a multimeric plasma protein that mediates platelet adhesion and platelet-platelet interactions [1]. VWF binds via its A3 domain to exposed subendothelial collagen at sites of vascular injury. Collagen-bound VWF tethers platelets to the vessel wall via transient interaction of its A1 domain with the platelet glycoprotein (GP)Ib-IX-V receptor complex [2]. Circulating VWF can only exert this function after conversion from its latent, globular conformation to an active conformation, in which the binding site for platelet GpIbα is exposed. Under physiological conditions, conversion to this active state is well regulated. Upon vascular injury, VWF immobilization to subendothelial collagen in conjunction with increased shear stress induce VWF unfolding [3], allowing for platelet-VWF interaction [4].

Various pathological conditions are associated with premature and/or excessive formation of VWF–platelet aggregates [5]. Von Willebrand disease (VWD) type 2B, for instance, is characterized by increased interactions between VWF and platelets, resulting from gain-of-function mutations (e.g. R1306W) in the VWF A1 domain [6]. Consequently, these patients lack high-molecular-weight VWF multimers and suffer from thrombocytopenia, clinically resulting in a bleeding phenotype [7]. In thrombotic thrombocytopenic purpura (TTP) patients, an acquired or inherited deficiency of the VWF cleaving protease ADAMTS13 results in accumulation of ultralarge (UL)-VWF multimers [8]. Clinically the result is a thrombotic phenotype caused by platelet-rich thrombi occluding the microvasculature [8]. Spontaneous VWF-platelet interaction in these conditions is indicative of the presence of VWF in its active conformation.

Several laboratory tests to assess VWF are available. The VWF antigen (VWF:Ag) assay is a quantitative assay that provides an overall level of VWF present in plasma, but yields no information concerning the quality of VWF in terms of its ability to bind platelets [9]. The ristocetin cofactor (VWF:RCo) assay is the most widely used method to assess the functional activity of VWF [10]. However, VWF:RCo assays are highly variable [11] and the need for ristocetin to activate VWF does not reflect the *in vivo* situation. A more recently developed assay uses recombinant GPIb fragments with two gain‐of‐function mutations that allow binding to VWF in the absence of ristocetin (VWF:GPIbM) [12]. Although this VWF:GPIbM assay eliminates the need for the non-physiological activator ristocetin, it is based on non‐physiological binding of VWF to a mutant receptor. Therefore, an immunosorbent assay to directly detect circulating VWF in its active conformation was previously developed [13]. This assay is based on a recombinant llama-derived antibody (AU/VWFa-11) that preferentially binds the GpIbα-binding conformation of the VWF A1 domain [13]. Using this assay, elevated active VWF levels were identified in plasma from patients with VWD type 2B [13], TTP [13], HELLP syndrome [14], systemic inflammatory response syndrome [15], antiphospholipid syndrome [16], diabetes [17], first ST-segment elevation myocardial infarction (STEMI) [18], sickle cell disease [19], malaria [20] and dengue [21]. Together, these findings have provided new insights into the presence of, as well as the mechanism behind increased active VWF in these pathological conditions. Moreover, quantification of active VWF has potential value for diagnostics, for example to differentiate VWD 2B from other types of VWD, to distinguish between forms of VWD 2B and to differentiate between patients with acquired and congenital TTP [13, 22]. However, there are no reports of an analytical validation of this assay. Therefore, we developed our own in-house immunosorbent assay to measure active VWF, also based on a llama-derived variable heavy chain antibody fragment (VHH) directed against a cryptic epitope in the A1 domain of VWF. The aims of the current study were to (1) characterize the analytical performance of this assay; (2) provide a reference interval for active VWF and (3) determine correlations between this assay and other, established VWF assays and a whole blood flow cytometric assay for platelet-VWF binding (Plt:VWF binding, adapted from others [23–25]).

## Materials and methods

### Ethics statement

The research complied with all the relevant national regulations, institutional policies and the tenets of the Helsinki Declaration, and has been evaluated by the Medical Ethical Committee (METC) of Maastricht University Medical Center/University of Maastricht (METC reference 152015). Participants gave full written informed consent.

### Reagents

Haemate P (CSL Behring, Pennsylvania, USA) VWF/FVIII concentrate (stock 12 mg/mL) was used as native human VWF in its globular conformation, referred to as ‘‘HVWF”. Recombinant VWF with the VWD type 2B R1306W (stock 17 μg/mL) or R1306Q (stock 55 μg/mL) mutation (produced as described previously [26]) was used as VWF constitutively in its active conformation. Polyclonal rabbit anti-VWF antibody conjugated to HRP was purchased from Dako (P0226, Glostrup, Denmark). Bovine serum albumin (BSA; A7906), SIGMAFAST *o*-Phenylenediamine dihydrochloride (OPD; P9187) and sulfuric acid (H_2_SO_4_; 339741) were all purchased from Sigma–Aldrich (Zwijndrecht, The Netherlands).

### Production of VHH

See S1 Methods for a detailed description of VHH production.

### Study population and plasma preparation

The study population consisted of 120 healthy individuals, aged 18–65 years, recruited between March 12, 2018 and May 17, 2018. Participants did not take any anti-coagulant or anti-platelet drugs for at least one week and did not have a history of thrombosis or bleeding. Analytical characterization of the assay was performed using residual, anonymized citrated plasma from the reference value study samples as well as residual, anonymized citrated plasma initially collected for routine laboratory diagnostics conducted at the Gelre Hospitals Apeldoorn, the Netherlands. Blood was collected by antecubital venepuncture into vacuum tubes (1 volume trisodium citrate 0.105M to 9 volumes blood) (BD Vacutainer System, Becton Dickinson, Franklin Lakes, USA). Cell counts in whole blood were performed with a Coulter Counter analyser (Beckman Coulter, Woerden, The Netherlands). Plasma was prepared by centrifugation at 2,840 g for 10 minutes, pipetting off the plasma fraction, and repeating centrifugation to obtain the platelet-poor plasma fraction, which was stored at -80°C.

### Active VWF immunosorbent assay

**Fig 1:**
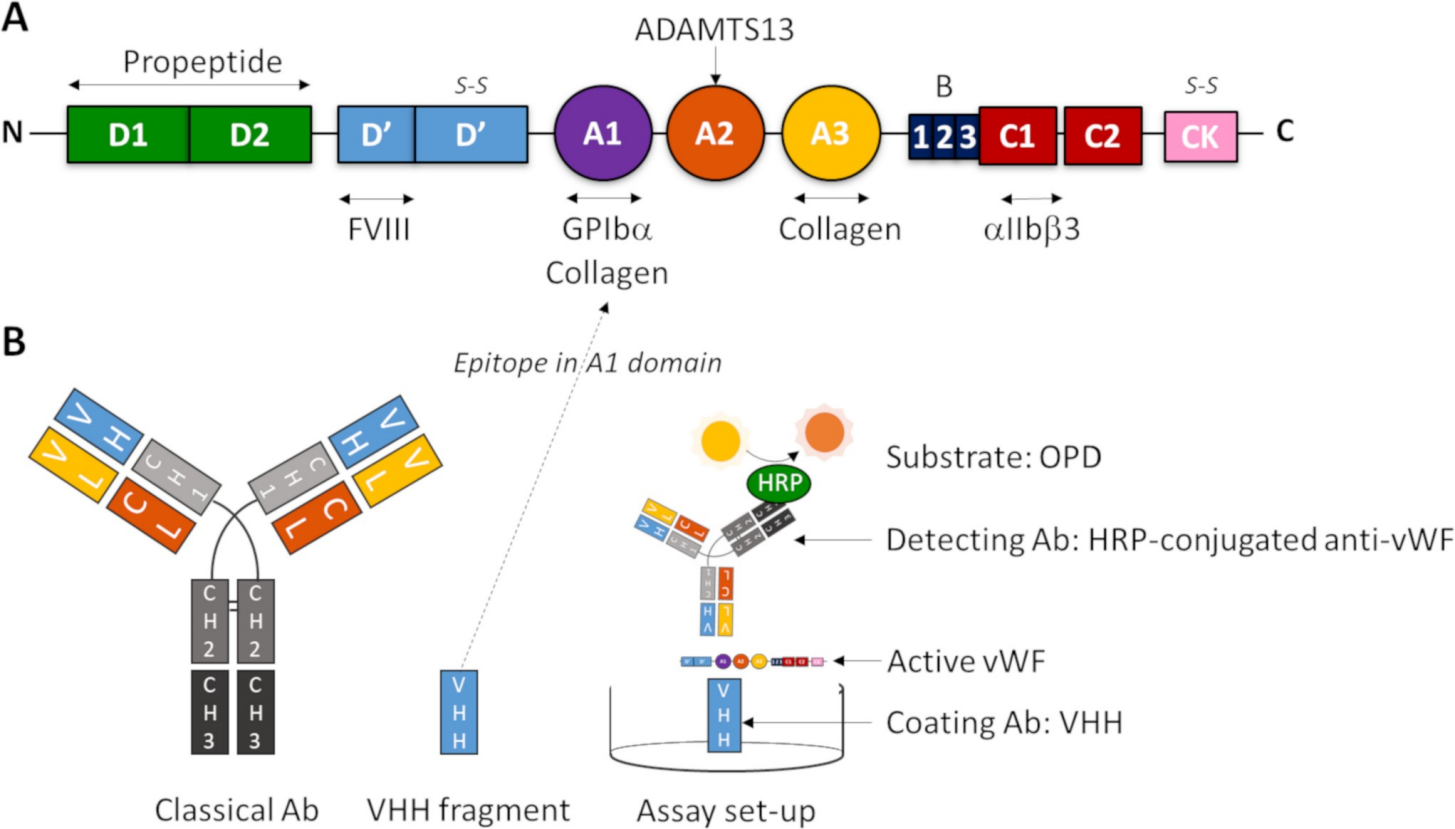
Scheme for measurement of active VWF. (A) The protein domain structure of mature VWF (adapted from Crawley et al.[27]). (B) The set-up of the immunosorbent assay described here, based on a variable heavy chain antibody fragment (VHH) directed against a cryptic epitope in the A1 domain of VWF as capture antibody, and a polyclonal rabbit anti-human VWF antibody conjugated with HRP as detecting antibody.

The active VWF assay is based on a llama-derived single-domain antibody (VHH) directed against a cryptic epitope in the A1 domain of VWF (Fig 1), which is only exposed upon unfolding of VWF [13]. Briefly, 96 wells microtiter plates (NUNC Maxisorp, Thermo Fisher Scientific, Waltham, USA) were coated overnight at 4°C with 1.98 μg/ml VHH against active VWF in a carbonate-bicarbonate coating buffer (pH 9.6), followed by a blocking step with 2% BSA in phosphate-buffered saline (PBS) for 45 minutes at room temperature (RT). After washing with 0.01% Tween-20 in PBS, plasma samples (diluted 1:20 in PBS/1% BSA) were incubated for 2 hours at RT. Following another wash-step, the wells were incubated with HRP-conjugated anti-VWF antibodies (1.2 μg/mL) in PBS/1% BSA for 2 hours at room temperature. Plates were then washed again before addition of SIGMAFAST OPD (Sigma). The reaction was stopped with 3 M sulfuric acid (H_2_SO_4_, Sigma). Optical densities (OD) were measured at 490 nm using an ELx808 Absorbance Microplate Reader (Biotek, Bad Friedrichshall, Germany). Results were normalized (%) to normal pooled plasma (NPP) on the same plate. This differs from the quantification method in earlier studies using the previous version of this assay, reporting the VWF activation factor [13]. The VWF activation factor was calculated by dividing the absorbance slope of the dilution curves for a patient sample by the slope of a NPP sample. We simplified the assay and reduced required sample volume by measuring one dilution of each plasma sample and expressing its active VWF level compared to that in NPP. For the NPP, blood from 10 healthy control donors was collected. After double centrifugation (2,840 g for 10 min), plasmas were pooled and aliquots of 1 ml were stored at -80°C until use in the active VWF ELISA. Of note, NPP contains only a very small amount of active VWF. If an individual has an active VWF level of 150%, he/she has 1.5 times more circulating active VWF than is present in the NPP, which is still a minute amount, but the current assay is sensitive enough to detect this difference.

### Assay performance studies

See S1 Methods for a detailed description of the assay performance studies.

### Established VWF parameters

VWF antigen (VWF:Ag) levels were determined using the Liatest VWF assay on an automated STA-R Max coagulation analyser (Diagnostica Stago, Leiden, the Netherlands). VWF ristocetin cofactor activity (VWF:RCo) was measured with the automated chemiluminescent HemosIL VWF:RCo assay (Werfen-Instrumentation Laboratory, Bedford, USA). VWF propeptide (VWFpp) was measured using the anti-human VWFpp MW1939 antibody pair and Tool Set 2 (Sanquin, Amsterdam, The Netherlands) [28] according to the manufacturer instructions, with the exception that VWFpp was expressed as a % of the level in NPP included on the same plate (due to limited number of available kits the recommended 8 step 2-fold serial dilution curve could not be included) VWF:GP1bM was measured using the INNOVANCE VWF Ac assay on a Sysmex CS-2500 instrument (Siemens Healthcare Diagnostics GmbH, Marburg, Germany) according to the manufacturer's protocol.

### Flow cytometric analysis of Plt:VWF binding

See S1 Methods for a detailed description of this assay, for which standardisation and a reference range were described previously [29].

### Statistical analysis

Outlier analysis was performed using Tukey’s hinges. One outlier in the active VWF values was detected (167.9%) but there was no evidence of an experimental error and this value was hence not excluded. Normality was tested using Q-Q plots and the Shapiro-Wilk test. None of the VWF parameters were normally distributed. Continuous variables were expressed as median and interquartile range (25%-75%). Reference intervals were obtained using nonparametric calculation, according to the latest CLSI guidelines [30], i.e. the 2.5^th^ percentile to the 97.5^th^ percentile of the distribution. Associations between variables were determined using Spearman’s rank correlation coefficient. Groups were compared using the Mann-Whitney U test for independent samples (continuous variables) or Chi-square test for categorical variables. Multiple linear regression was used to adjust for the effect of differences in VWF:Ag levels on associations between (1) blood group and VWF:RCo, VWF:GP1bM and Plt:VWF binding, and (2) sex and active VWF levels. A p-value of 0.05 was considered statistically significant for all comparisons. Analyses were performed using Statistical Package for Social Sciences (SPSS Incorporate, Chicago, USA) version 25. Figures were prepared using GraphPad Prism version 5.00 (Graphpad Software, San Diego, USA).

## Results

### VHH specifically detects active VWF

**Fig 2:**
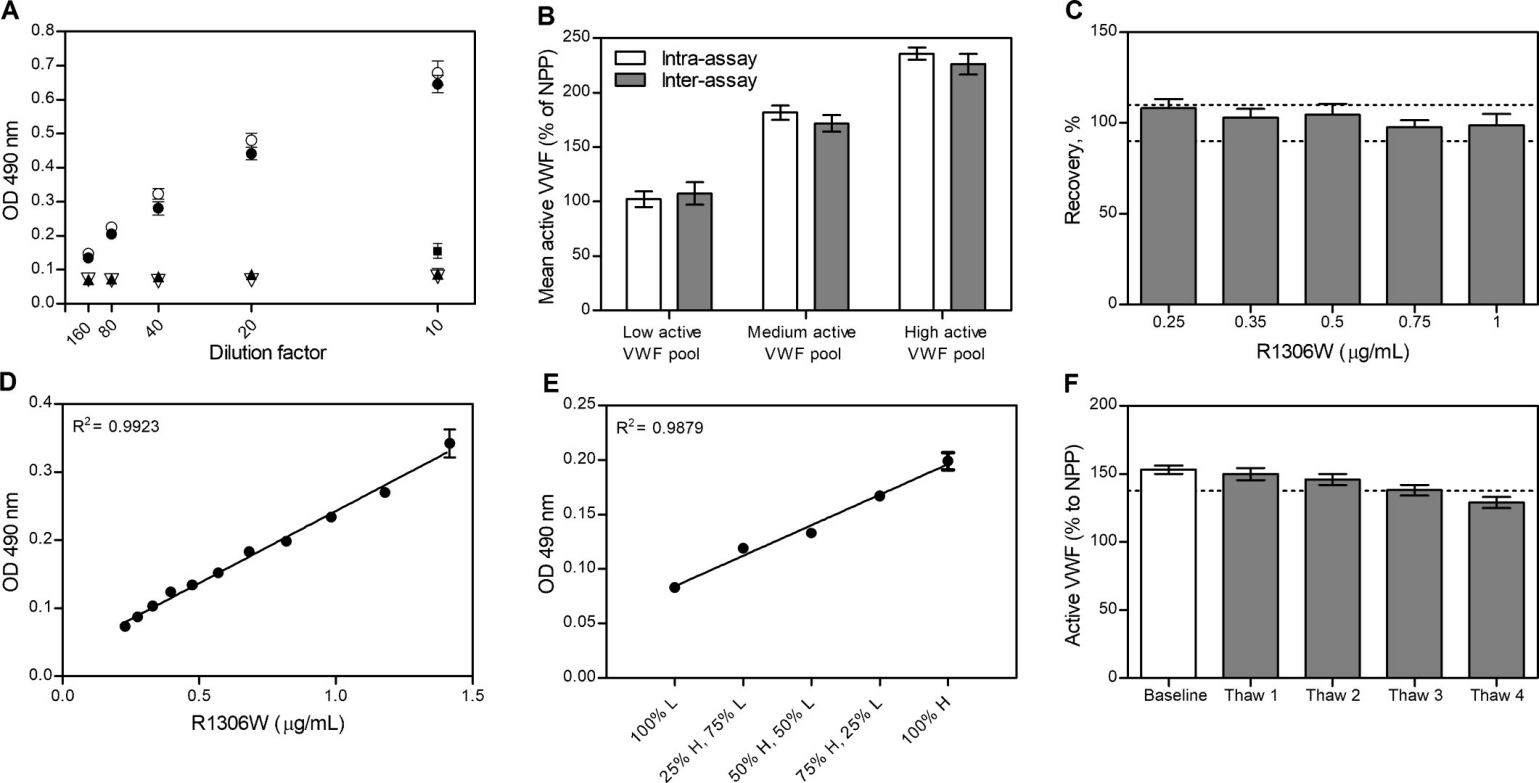
Analytical characteristics of the active VWF assay. (A) Binding of the VHH to R1306W VWF (●), R1306Q VWF (○), HVWF (σ), VWF in NPP under static conditions (π) and NPP after vortexing to simulate shear stress (▪, only 10x diluted). Dilution factors are indicated at the x-axis. (B) Mean active VWF levels in plasma pools (with low (91–110%), medium (150–200%) and high (>220%) active VWF levels) with corresponding SDs for 20 replicates on a single plate on the same day (intra-assay, white bars) or on 20 separate days (inter-assay, grey bars). (C) Recovery (%) of increasing levels of R1306W VWF spiked to plasma pool. The acceptance range of 90–110% of expected values is indicated by dotted lines. (D) Assay response (OD at 490 nm) for a 12-step, 1.2-fold dilution series of plasma spiked with R1306W. Linear regression parameters (±SD) are: slope 0,21 ± 0.006, intercept 0.03 ± 0.005, SD of residuals 0.007. (E) Low (L) and high (H) active VWF plasma pools were mixed in varying proportions to assess dilutional linearity. (F) Stability of active VWF in a plasma pool at baseline and after multiple freeze-thaw steps (Thaw 1–4). The acceptance criterium of <10% analyte loss is indicated by the dotted line. Data represent the mean±SD (n = 3) unless otherwise stated.

To test the specificity of the VHH for the active conformation of VWF, its binding to HVWF, R1306W VWF, R1306Q VWF, and VWF in NPP was investigated (Fig 2A). The VHH neither interacted with HVWF nor VWF in NPP, as apparent from the weak (background) signal at 490 nm. In contrast, binding of the VHH to constitutively active R1306W and R1306Q VWF resulted in a strong dose-dependent signal. To confirm that the VHH also recognizes native VWF that is activated by high shear stress we also tested vortexed NPP. As a result, the VHH interacted with the shear-unfolded VWF in NPP. Thus, the VHH specifically recognizes VWF in its active conformation.

### Analytical performance of active VWF assay

We proceeded with the analytical characterization of the assay, summarized in Table 1 and depicted in Fig 2B–2F. Precision studies revealed intra-assay CVs of 2.4–7.2% and inter-assay CVs of 4.1–9.4%, depending on measurand level (Fig 2B). Spiked R1306W VWF could be accurately measured in a plasma matrix at concentrations as low as 0.25 μg/mL, with observed levels well within 90–110% of the expected levels (Fig 2C). The assay was linear (R^2^>0.99) in almost the entire range of dilutions of plasma spiked with R1306W (Fig 2D and Table 1). Recovery of active VWF in mixes of low (L) and high (H) plasma pools was within the pre-defined acceptable limits of 90–110%. Moreover, the curve fitted through the 5 points for these mixes (expected OD based on levels in individual pools versus observed) was linear (R^2^ = 0.99) (Fig 2E). The limit of quantitation (LoQ) was 101% (corresponding to an OD of 0.081). The limit of blank (LoB) and limit of detection (LoD) were 80.0% (OD 0.060) and 89.1% (OD 0.067), respectively. Active VWF in citrated plasma was stable for at least 2 months (longest time tested) at -80°C (Table 1). Importantly, freeze-thawing up to 3 times slightly decreased the detected active VWF, whereas more frequent freeze-thawing resulted in loss of analyte exceeding the criterion of 10% (Fig 2F).

**Table 1:** Analytical performance characteristics of the active VWF ELISA assay.

Parameter	Assay performance	
**Precision**	*Intra-assay (Mean [SD;%CV])*	*Inter-assay (Mean [SD;%CV])*
	L 102% [7.3%; 7.2%]	L 107% [10.1%; 9.4%]
	M 182% [6.6%; 3.7%]	M 172% [7.6%; 4.4%]
	H 235% [5.7%; 2.4%]	H 226% [9.2%; 4.1%]
**Accuracy**	*Spiked 1*.*0 μg/mL*	
	[E]_OD_ 0.233 - [O]_OD_ 0.230	[E]_%_ 333 - [O]_%_ 329—Rec% 99
	*Spiked 0*.*75 μg/mL*	
	[E]_OD_ 0.171 - [O]_OD_ 0.175	[E]_%_ 250 - [O]_%_ 245—Rec% 98
	*Spiked 0*.*5 μg/mL*	
	[E]_OD_ 0.117 - [O]_OD_ 0.122	[E]_%_ 167 - [O]_%_ 175—Rec% 105
	*Spiked 0*.*35 μg/mL*	
	[E]_OD_ 0.093 - [O]_OD_ 0.101	[E]_%_ 140 - [O]_%_ 144—Rec% 103
	*Spiked 0*.*25 μg/mL*	
	[E]_OD_ 0.069 - [O]_OD_ 0.076	[E]_%_ 100 - [O]_%_ 108—Rec% 108
**Linearity**	**Spiked with R1306W aVWF**	
	Assessed: OD 0.073–0.635	(90.1%– 787.2% of NPP)
	Linear range: OD 0.073–0.342	(90.1%– 424.5% of NPP)
	Linearity curve through points:	R^2^ = 0.9923
	**Dilution of L with H pool**	
	Range: OD 0.083–0.199	
	*Mix content—aVWF mean (SD)*	*- % of expected*
	100% H / 0% L—236 (5.5)	- n.a.
	75% H / 25% L—208 (2.1)	- 103
	50% H / 50% L—165 (1.9)	- 97
	25% H / 75% L—147 (1.9)	- 108
	0% H / 100% L—103 (2.3)	- n.a.
	Linear fit [O]_OD_ vs [E]_OD_:	R^2^ = 0.9879
	*OD—% of NPP*	
**LoQ**	0.081–101.2	
**LoB**	0.060–80.0	
**LoD**	0.067–89.1	
**Stability**	Baseline aVWF: 153% (SD 2.7%)	
	**Repeated freeze-thawing**	**Recovery extended storage**
	*Thaw #—mean (SD)—*% Rel. to BL	*t—mean (SD)*—% Rel. to BL	
	1–150 (3.8) - 98	24h -153 (2.6) - 100	
	2–146 (3.2) - 95	48h - 152 (3.3) - 100	
	3–138 (4.4) - 90	7d- 150 (4.0) - 98	
	4–129 (3.2) - 84	1m - 146 (3.2) - 95	
		2m - 147 (2.3) - 96	

SD, standard deviation; CV, coefficient of varation; L, low; M, medium; H, high; E, expected; O, observed; OD, optical density; [E]_%_/[O]_%_, expected and observed active VWF (aVWF) level, as a % of NPP; LoQ, limit of quantitation; LoB, limit of blank; LoD, limit of detection; NPP, normal pooled plasma; Rel. to BL, relative to baseline value, calculated as 100x(observed value/baseline), expressed as %; t, timepoint; h, hours; d, days; m, month.

### Reference interval and inter-individual variation for (active) VWF

Demographic data and blood counts of 120 healthy individuals are summarized in Table 2. Hemoglobin levels, hematocrit and red blood cell counts were significantly higher in male compared to female subjects.

**Table 2:** Demographic and laboratory results of healthy donors in reference interval study.

Parameter	All	Male (n = 60)	Female (n = 60)	p-value
**General**				
Age (years)	31 (25–44.5)	29 (25–39)	32.5 (26–50)	ns
OC use (%)	15	-	15 (25%)	-
**Haematological**				
Blood group^a^ (%)	O n = 26 (40%)	n = 12 (41%)	n = 14 (39%)	ns
	A n = 29 (45%)	n = 12 (41%)	n = 17 (47%)	
	B n = 9 (14%)	n = 5 (17%)	n = 4 (11%)	
	AB n = 1 (1%)		n = 1 (3%)	
Platelets (x10^9^/L)	233 (208–270)	229 (207–257)	244 (216–284)	ns
WBC (x10^9^/L)	5.2 (4.6–6.0)	5.2 (4.6–5.8)	5.3 (4.7–6.2)	ns
RBC (x10^12^/L)	4.5 (4.2–4.7)	4.6 (4.5–4.8)	4.3 (4.1–4.5)	<0.0001
Hb (mmol/L)	7.9 (7.5–8.4)	8.3 (8.0–8.6)	7.6 (7.2–7.9)	<0.0001
Hct (L/L)	0.39 (0.37–0.41)	0.40 (0.39–0.41)	0.37 (0.35–0.39)	<0.0001
MPV (fL)	7.5 (7.1–8.2)	7.4 (7.1–8.2)	7.6 (7.2–8.3)	ns

OC, oral contraceptives; freq, frequency; WBC, white blood cell count; RBC, red blood cell count; Hb, haemoglobin; Hct, haematocrit; MPV, mean platelet volume.

^a^ Blood group known for 65 individuals (36 female, 29 male), percentages indicate proportions for each blood group from total individuals with known blood group. Medians and interquartile ranges (25–75%) are given unless otherwise indicated.

The distribution of active VWF (S1 Fig) in this population showed a clear right-skewedness, with the majority of values approximating that in NPP (100%). The corresponding reference interval for active VWF was 91.6–154.8%. Reference intervals for VWF:Ag, VWF:RCo, VWF:GP1bM, VWFpp and Plt:VWF binding were 65.1–189.9%, 47.0–161.5%, 58.3–189.7%, 73.3–205% and 1.4–15.6% respectively (Table 3). Moreover, active VWF had a considerably lower inter-individual variation (15.1%) than all other VWF parameters.

**Table 3:** Reference intervals for active VWF and other VWF parameters in 120 healthy individuals.

Parameter	Mean	SD	%CV	Median	IQR	Ref. interval 2.5%-97.5%	Min	Max
VWF:Act (%)	115.4	17.4	15.1	112.9	25.1	91.6–154.8	91.0	167.9
VWF:Ag (%)	115.1	32.2	28.0	104.5	39.8	65.1–189.9	40.0	218.0
VWF:RCo (%)	103.9	32.9	31.7	102.6	48.1	47.0–161.5	20.8	243.4
VWF:GP1bM (%)	112.9	32.9	29.1	110.6	43.1	58.3–189.7	41.3	229.5
VWFpp (%)	121.8	23.1	24.1	121.2	32.1	73.3–188.7	60.3	210.3
Plt:VWF (%)	6.9	3.2	46.4	6.4	3.8	1.4–15.6	0.9	18.0

%CV, inter-individual variation expressed as coefficient of variation, calculated as (SD/Mean)*100; IQR, interquartile range, calculated as 75^th^– 25^th^ percentile; Ref. interval, reference interval, calculated as the 2.5^th^ percentile to the 97.5^th^ percentile of the distribution; VWF:Act, active VWF, % of level in NPP. VWF:Ag, VWF antigen; VWF:RCo, VWF ristocetin cofactor activity; VWF:GP1bM, VWF binding to gain-of-function GP1b fragments; VWFpp, VWF propeptide, % of level in NPP; Plt:VWF, platelet-VWF binding, % of signal for beads.

### Mutual correlations of VWF parameters

**Fig 3:**
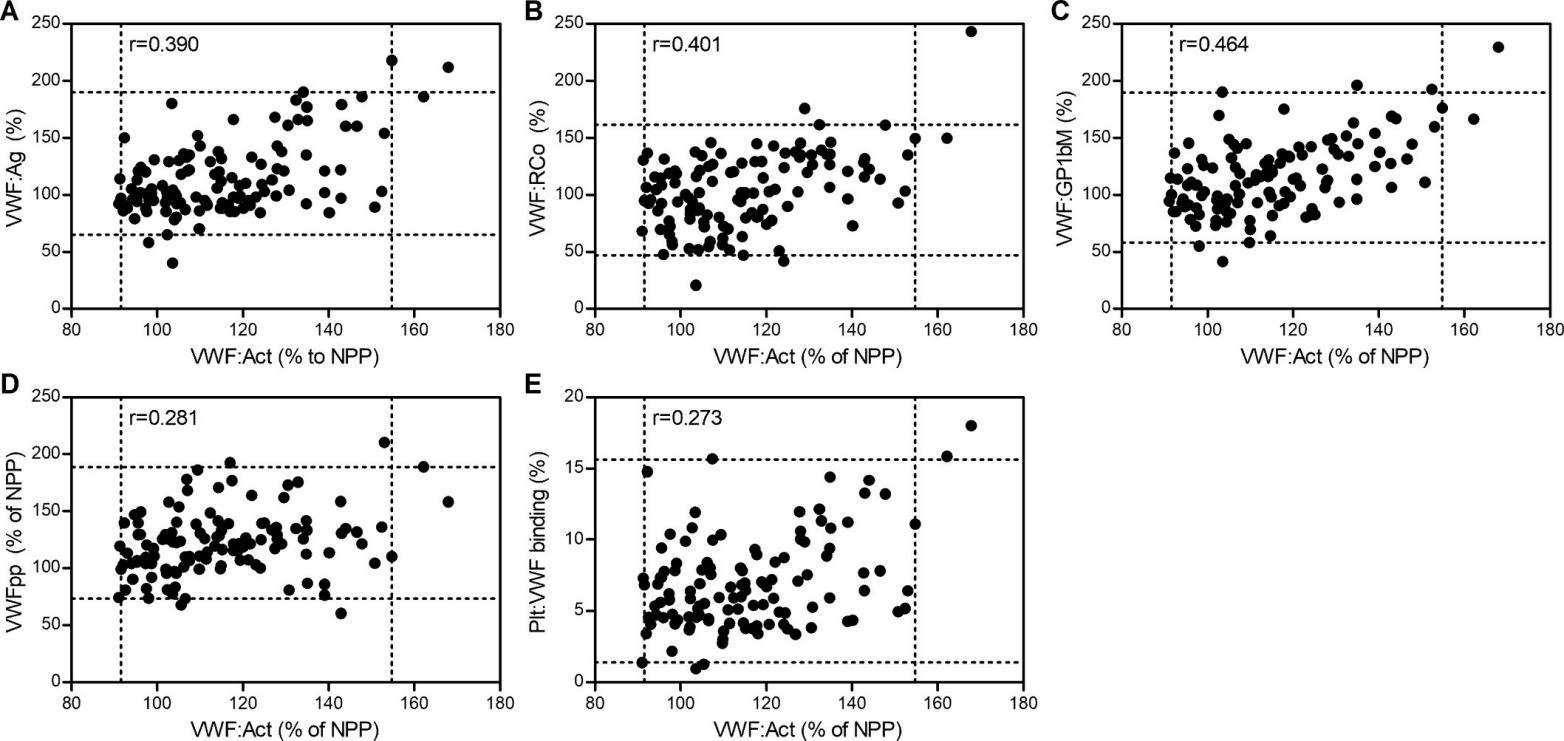
Correlation of active VWF with other VWF parameters. Scatterplots and corresponding Spearman rank correlations (r) between active VWF assay (VWF:Act) and (A) VWF:Ag, (B) VWF:RCo, (C) VWF:GP1bM, (D) VWFpp and (E) Plt:VWF binding. Dotted lines delineate reference intervals determined in this study, with the centre square containing 95% of values. Spearman rank correlation coefficients (r) are indicated in the upper left corner of each panel.

A variety of assays may be performed for the assessment of VWF in plasma, each quantifying a different form of VWF (globular native VWF:Ag, active VWF, VWFpp) or providing qualitative information on its function (VWF:RCo, VWF:GP1bM, Plt:VWF binding). Significant correlations were observed between all these VWF parameters in our study population. Active VWF levels were significantly and positively correlated with VWFpp (r = 0.281, p = 0.005), Plt:VWF binding (0.273, p = 0.003), VWF:Ag levels (r = 0.390, p<0.001), VWF:RCo (r = 0.401, p<0.001) and the highest correlation was found with VWF:GP1bM (r = 0.464, p<0.001) (Fig 3 and S1 Table). Overall, the highest degree of correlation (p<0.001) existed between VWF:GP1bM activity and VWF-platelet binding as measured by the Plt:VWF binding assay. There were also strong associations between these assays and the other two functional assays for active VWF and VWF:RCo, and between the functional assays and VWF:Ag (S1 Table).

### VWF parameters related to clinical parameters

Active VWF levels correlated with gender (r = 0.21, p = 0.019) and were significantly (p = 0.033) higher in male (median 115.2%, IQR 105.6–127.4%) compared to female subjects (107.1%, IQR 97.5–126.1%). Therefore, sex-specific reference intervals were calculated, but these did not differ considerably (93.3–158.5% for men, 91.2–159.7% for women), and corresponding confidence intervals overlapped (S2 Fig). VWF:Ag levels were also significantly (p = 0.044) higher in male (median 112.5%, IQR 95.5–135.3%) compared to female subjects (median 99.5%, IQR 91.0–122.5%). Adjustment for VWF:Ag levels using multiple linear regression strongly reduced the association between active VWF and sex (p = 0.047 to p = 0.289). Active VWF did not correlate with age, whereas significant positive correlations of VWF:Ag levels (r = 0.236, p = 0.009) and VWF:RCo (r = 0.181, p = 0.048) with age were observed (S3 Fig). No association between active VWF levels and blood group was found (r = 0.196, p = 0.118), while VWF:Ag levels (r = 0.296, p = 0.018), VWF:RCo (r = 0.299, p = 0.017), VWF:GP1Bm (r = 0.280, p = 0.025) and Plt:VWF binding (r = 0.293, p = 0.021) were significantly lower in O compared to non-O blood group subjects (S4 Fig). However, in multiple linear regression analysis adjustment for VWF:Ag levels eliminated the significant association between VWF:RCo (p = 0.270), VWF:GP1bM (p = 0.297) and Plt:VWF binding (p = 0.612) with blood group.

## Discussion

Here, we describe an immunosorbent assay for the reliable, accurate and specific quantification of active VWF in citrated plasma. We established its reference interval in a healthy population and we demonstrated a clear association of active VWF levels with four clinically established VWF assays and a flow cytometric VWF-platelet binding assay.

The active VWF assay fulfilled our pre-defined analytical requirements. First, the assay was highly specific for active VWF, as only the two recombinant VWF proteins that are constitutively in their active conformation were detected and no cross-reactivity with native (NPP) and purified HVWF was observed. This is in accordance with findings by Hulstein et al [13]. Of note, serial dilutions of NPP did not substantially increase the OD since NPP only contains minute amounts of active VWF. Reproducibility was adequate, with values below 10% for both the intra- and inter-assay CV, comparable with those found by others [13, 18]. All values obtained from the population of healthy individuals were above the LoD (89.1%), whereas 26 (22%) values were below the LoQ of 101%. This is not necessarily a limitation, since high active VWF levels are much more relevant as they may induce thrombosis or bleeding. Lastly, active VWF showed good stability in samples stored at -80°C, and a maximum of 3 freeze-thawing steps should be allowed to prevent analyte loss of more than 10%. This effect of freeze-thawing may be explained by an increased proportion of small molecular weight fragments, as was observed previously [31]. Taken together, these data demonstrate the robust analytical performance of this assay.

In order to identify elevated active VWF levels in patient samples, it is essential to establish a reference interval in healthy individuals. To this end, we determined active VWF levels in 120 healthy individuals, as recommended by CLSI guidelines [30]. The reference range shows a right-skewed distribution, with most individuals having very low active VWF levels, similar to that in NPP (set to 100%). The right skew indicates that slightly more active VWF is present in native plasma of some healthy individuals, and these small differences could be detected due to the high sensitivity of our assay. The reference interval includes the majority of active VWF levels reported by others, although values below the LoD of the current assay were previously reported [16, 20, 21, 32]. This difference may be attributed to an alternative calculation method for the VWF ‘activation factor’ (see limitations below) [13]. Of note, the number of healthy controls in previous studies was much lower than 120, ranging from nine [13, 14] to fifty-nine [17].

In addition, we determined VWF:Ag, VWF:RCo, VWF:GP1bM, VWFpp and Plt:VWF binding in these 120 samples.

Increased active VWF can potentially have major impact on the risk of bleeding and/or thrombosis, as exemplified by VWD type 2B and TTP [13]. Therefore, regulatory mechanisms, for instance the natural inhibitor β2GPI, are keeping active VWF levels low in healthy individuals [16]. The inter-individual variation in active VWF levels (15.1%) was nearly half of that in VWF:Ag levels and the majority of active VWF values were close to the level in NPP (100%). The observed inter-individual variation for VWF:Ag was similar to the previously reported inter-individual biologic variation for VWF [33].

We identified significant positive correlations between all VWF parameters (S1 Table). With regard to active VWF, the highest correlation was found with two other activity assays, namely VWF:RCo and VWF:GP1bM as expected based on assay principles. Active VWF circulates in a GPIbα-binding conformation and will therefore contribute to the chemiluminescent signal in the VWF:RCo assay, generated when VWF (either active VWF already present or VWF activated by ristocetin) binds to microparticles coated with GPIbα. Similarly, in the VWF:GP1bM assay only active VWF can bind spontaneously to the microparticle-(mutant) GP1b complex, inducing agglutination that is measured turbidimetrically. Whereas the VWF:GP1bM and VWF:RCo assays are much more widely established, advantages of the active VWF ELISA assay are that neither non-physiological additives (ristocetin or mutant GP1b) nor specialized instruments are required. Moreover, active VWF correlated well with VWF:Ag levels (r = 0.390), showing that with increasing VWF the amount of circulating VWF in its active, unfolded conformation was also higher. This is in accordance with correlations found between VWF activation factor and VWF:Ag in previous studies in patients with a first STEMI (r = 0.58) [18] and malignant hypertension (r = 0.62) [34]. The stronger correlation in these studies may be explained by reporting the activation factor, which corrects for differences in VWF:Ag levels. Moreover, active VWF correlated with VWFpp. Relatively increased VWFpp is commonly used as a marker for acute endothelial cell activation, while relatively high VWF:Ag levels are considered a marker for more chronic endothelial cell activation [28]. VWF in endothelial cells is likely in its active conformation, as freshly secreted VWF is able to interact with the platelet GpIb–IX–V complex [5]. Thus, it can be postulated that circulating active VWF was secreted from endothelial cells (hence correlating with VWFpp and VWF:Ag levels), but escaped ADAMTS-13 proteolysis.

In our healthy population, active VWF and VWF:Ag levels were significantly higher in men than in women In contrast to the activation factor calculation method [13], our calculation of active VWF levels does not correct for differences in VWF:Ag levels. Therefore, sex-related differences in active VWF levels have not been reported in previous studies. Whereas the majority of the literature on the influence of sex (gender) on VWF:Ag reports no differences [9, 35], Conlan et al. reported higher VWF:Ag levels in female compared to male subjects [36], while Campos et al. found higher VWF:Ag levels in men than in women [37]. In addition, both older age and non-O blood group were associated with higher VWF:Ag levels, as described in literature [9, 38–41]. VWF:RCo, VWF:GP1bM and Plt:VWF binding were also higher in individuals with a non-O blood group, but correction for VWF:Ag levels rendered these associations non-significant.

A possible limitation of the current assay is that it expresses the amount of active VWF as a percentage, normalized to values in NPP. We consider this a more reliable approach than expressing the absolute concentration with regard to a monomeric VWF A1 domain, because one VHH binds active VWF multimers with different sizes [42] and hence variable numbers of VWF epitopes. The assay cannot adjust for variation in the multimeric composition between individuals. In addition, in order to calculate the concentration of active VWF, we would need to assume that the VHH recognizes mutation-induced activated VWF (e.g. R1306W) similarly as non-mutated (activated) VWF and as active VWF in a variety of conditions. Because we are uncertain of these assumptions, we propose that expressing active VWF as a percentage of NPP is a more reliable calibrator for the moment. However, the normalized result makes it more difficult to standardize the assay, and the reported reference interval may have been slightly different using another batch of NPP. Given the small inter-individual variation in active VWF in healthy individuals and the large increases observed in several pathologies [5], we do not expect minor differences in NPP active VWF levels to cause wrong classification of active VWF in a sample as pathological.

### Conclusions

Previously, it has been shown that several pathological conditions are associated with strongly increased active VWF levels [5]. The underlying mechanism can likely be attributed to (a combination of) three causes: (1) changes in the (conformation of) VWF itself (e.g. VWD type 2B [13]), (2) changes in endothelial secretion (e.g. malaria [20], diabetes [17]), processing or clearance of VWF (e.g. TTP [13], HELLP [14]) or (3) increased shear stress (e.g. mild aortic stenosis [32], first STEMI [18]). Increased circulating active VWF levels may also be present in other conditions associated with infection and/or inflammation, for instance in chronic kidney disease (submitted). Moreover, using the current assay, we found that active VWF levels in healthy individuals increase following strenuous exercise [43]. Quantification of active VWF in a variety of diseases could provide valuable pathophysiological insight for diagnosis and development of new treatment strategies. The current validation of an immunosorbent assay for active VWF and the establishment of a reference interval will facilitate future studies investigating the role of active VWF in health and disease.

## Supporting information

S1 Fig Distribution of active VWF in a healthy population. Citrated plasma samples obtained from 120 healthy subjects were measured with the active VWF assay. Limit of detection (LoD) and Limit of Quantitation (LoQ) are indicated. The frequency distribution shows skewness to the right.
(TIF)

S2 Fig Effect of gender on active VWF levels. Active VWF levels (normalized to levels in NPP) were determined in plasma of 120 healthy volunteers, and are shown here for males (n = 60) and females (n = 60). Median and IQR are indicated. The area delineated by the dotted lines represents the reference interval of the total population (2.5 percentile–97.5 percentile). Active VWF levels were significantly (p = 0.033) higher in males compared to females, but adjustment for VWF:Ag levels abolished the association between active VWF and gender.
(TIF)

S3 Fig VWF:Ag and VWF:RCo correlate significantly with age. Scatterplot and corresponding Spearman rank correlation (r) between VWF:Ag (a) and VWF:RCo (b) with age. Dotted lines delineate reference intervals for VWF:Ag and VWF:RCo determined in this study.
(TIF)

S4 Fig Effect of blood group O and non-O on VWF parameters. VWF:Ag (A), VWF:RCo (B), VWF:GP1bM (C) and Plt:VWF binding (D) were determined in plasma of 120 healthy volunteers, and are shown here for individuals with non-O and O blood group. Median and IQR are indicated. The areas delineated by the dotted lines represent the reference intervals (2.5 percentile–97.5 percentile). Statistical significance of differences in VWF parameters between O and non-O subjects were tested by Mann-Whitney U test. *, p<0.05.
(TIF)

S1 Table Spearman rank correlations between VWF assays. Values represent Spearman rank correlation coefficients with corresponding significance: **, p value <0.01. VWF:Act, active VWF; VWF:Ag, VWF antigen; VWF:RCo, VWF ristocetin cofactor activity; VWF:GP1bM, VWF binding to gain-of-function GP1b fragments; VWFpp, VWF propeptide; Plt:VWF, platelet VWF binding.
(DOCX)

S1 Methods Methodology for VHH production, assay performance studies and flow cytometric analysis of platelet-VWF binding. (DOCX)

S1 Database Database containing all raw data underlying Figs 1–3, Tables 1–3, S1–S4 Figs and S1 Table. (XLSX)
