# Supplementary material for: Analytical characterization and reference interval of an enzyme-linked immunosorbent assay for active von Willebrand factor
Source: PLoS One. 2019 Feb 13;14(2):e0211961. doi: 10.1371/journal.pone.0211961 (PMC6373957; doi:10.1371/journal.pone.0211961)
Supplement: S1 Table — Values represent Spearman rank correlation coefficients with corresponding significance: **, p value <0.01. VWF:Act, active VWF; VWF:Ag, VWF antigen; VWF:RCo, VWF ristocetin cofactor activity; VWF:GP1bM, VWF binding to gain-of-function GP1b fragments; VWFpp, VWF propeptide; Plt:VWF, platelet VWF binding. (DOCX) [file pone.0211961.s005.docx]

**S1 Table. Spearman rank correlations between VWF assays**

| **Assay** | **VWF:Act** | **VWF:Ag** | **VWF:RCo** | **VWF:GP1bM** | **VWFpp** | **Plt:VWF** |
| --- | --- | --- | --- | --- | --- | --- |
| **VWF:Act** |  |  |  |  |  |  |
| **VWF:Ag** | 0.390** |  |  |  |  |  |
| **VWF:RCo** | 0.401** | 0.617** |  |  |  |  |
| **VWF:GP1bM** | 0.464** | 0.669** | 0.564** |  |  |  |
| **VWFpp** | 0.281** | 0.457** | 0.442** | 0.404** |  |  |
| **Plt:VWF** | 0.273** | 0.636** | 0.619** | 0.679** | 0.396** |  |

Values represent Spearman rank correlation coefficients (r) with corresponding significance: **, p value <0.01. VWF:Act, active VWF; VWF:Ag, VWF antigen; VWF:RCo, VWF ristocetin cofactor activity; VWF:GP1bM, VWF binding to gain-of-function GP1b fragments; VWFpp, VWF propeptide; Plt:VWF, platelet VWF binding.
