# Supplementary material for: Analytical characterization and reference interval of an enzyme-linked immunosorbent assay for active von Willebrand factor
Source: PLoS One. 2019 Feb 13;14(2):e0211961. doi: 10.1371/journal.pone.0211961 (PMC6373957; doi:10.1371/journal.pone.0211961)
Supplement: S1 Methods — (DOCX) [file pone.0211961.s006.docx]

# **S1 Methods**

**Production of VHH**

VWF A1 domain specific VHH antibodies (MW 16.4 kDa) were commercially produced by U-Protein Express BV (Utrecht, the Netherlands). Briefly, HEK293E-253 cells were transfected with endotoxin-free maxiprep DNA for the desired sequence of the anti-VWF VHH (based on the sequence provided by [1]). Six days post-transfection conditioned medium containing recombinant protein was harvested by low-speed centrifugation (10 minutes, 1000 g) followed by high-speed centrifugation (10 minutes, 4000g) and immobilized metal affinity chromatography (IMAC) purification. The VHH was further purified by gel filtration using a Superdex75 26/600 column. The resulting VHH-containing fractions were sterilized by filtration over a 0.22 μm syringe filter and stored at 4 ▫C.

## **Assay performance studies**

**Specificity**

The assay was performed as described under ‘’Active VWF immunosorbent assay’’ in the Methods section. Normal pooled plasma (NPP, static or vortexed at 2,500 rpm for 10 minutes), HVWF, R1306W VWF or R1306Q VWF (all 1.7 ug/mL), were serially diluted (except for vortexed NPP, only measured at 1:10 dilution) with dilution buffer (PBS/1% BSA) and incubated in the plate coated with VHH at RT for 2 hours, followed by detection steps as described.

**Precision**

Pools of citrated plasma samples were prepared based on previously measured active VWF levels: the ‘’low’’ (L) pool contained samples with active VWF levels between 91 and 110% (mean 102%), the ‘’medium’’ (M) pool contained samples with active VWF levels between 150 and 200% (mean 182%) and the ‘’high’’ pool samples with active VWF levels >220% (mean 235%). These ‘’cut-offs’’ were arbitrarily chosen, to cover the range of relevant values, and they do not have clinical meaning. Intra-assay (within run) precision was assessed by repeated (n=20) measurements of these plasma pools active VWF in one plate on the same day. Inter-assay (between run) precision was determined by duplicate measurements of the same plasma pools on 20 different days, within 2 months. Acceptance criteria were CV ≤10% for intra-assay precision and CV ≤15% for inter-assay precision.

**Accuracy**

Accuracy was assessed by adding known concentrations of recombinant R1306W VWF (stock 17 μg/mL, concentrations 1.0, 0.75, 0.5, 0.35 or 0.25 μg/mL R1306W VWF) or dilution buffer (control) to NPP. Recovery (Rec%) was calculated based on the observed OD (O_OD_) and expected OD (E_OD_) as follows: Rec% = (O_OD_/E_OD_)*100% and was considered acceptable between 90% and 110%.

**Linearity**

Linearity was determined by two serial recovery studies. First, for spiking with recombinant R1306W VWF, a pool of plasma with low active VWF was prepared. Half of this low active VWF pool was spiked with recombinant R1306W VWF (maximal 10 v/v%, not higher to minimize matrix dilution effects) to obtain a high active VWF pool. The high active VWF sample pool was then diluted with the remaining low active VWF sample pool to create a 12 steps 1.2-fold dilution series. Secondly, to determine the linearity when diluting native plasma samples, a high pool (active VWF >200% of NPP, as determined in previous experiments) was diluted with a low pool (active VWF <120%, as determined in previous experiments) in 5 steps: 100% of each and 3 mixes of 75%/25%, 50%/50% and 25%/75%. For both dilution experiments three replicates per dilution step were measured in 1 run.

**Limits**

The limit of quantitation (LoQ) was determined using 11 citrated plasma pools over a range of concentrations (114 – 248%), each measured in duplicate over 10 days. The LoQ was extrapolated from the binomial curve through the points in the plot of mean active VWF (x-axis) versus inter-assay CV% (y-axis) as the mean active VWF level at which the inter-assay CV was 20%. The limit of blank (LoB) was determined by measuring a blank sample (dilution buffer) 60-fold and calculated as LoB=mean_blank_+1.645*SD_blank_. The limit of detection (LoD) was determined based on the LoB and 60 test replicates of a sample known to contain a low concentration of analyte (also used for intra-assay precision as ‘’low/L’’ pool). LoD was calculated as LoD=LoB+1.645(SD_low sample_).

**Stability**

We performed a stability study limited to the conditions used for (the validation of) this assay, i.e. with the aims to assess the (1) effect of repeated freeze/thawing and (2) stability during storage at -80°C. To determine freeze/thaw stability, a plasma pool with medium active VWF level (152.9%) was stored frozen at −80 °C and was measured in triplicate with the immunosorbent assay after being subjected to four freeze-thaw cycles during one day. Furthermore, at day 1, day 2, day 7, 1 month and 2 months aliquots of this pool were thawed and active VWF was determined in triplicate. Mean recovery of active VWF expressed in absolute values (i.e., absolute recovery, expressed as active VWF in % of NPP) and in percent of the baseline value (i.e., relative recovery) was calculated. The acceptance criterion was ±10% difference from the original concentration.

## **Flow cytometric analysis of VWF:platelet binding**

Test strips for three test conditions (no agonist control, 1.2 mg/mL ristocetin and positive beads control [PC]) were prepared in advance and stored at -20°C. The reaction mixtures with a total volume of 20 μl consisted of 2 μl FITC-conjugated anti-VWF antibodies (CL7616F, Cedarlane, Burlington, USA) and 0.5 μl APC-conjugated anti-CD41a antibodies (559777, BD Biosciences, San Jose, USA) with or without agonist in HEPES-buffered saline (HBS, 10 mmol/L HEPES, 150 mmol/L NaCl, 1 mmol/L MgSO_4_, 5 mmol/L KCL, pH 7.4). The PC tube only contained 2 μl FITC-conjugated anti-VWF antibodies in HBS.

After venipuncture, whole blood was kept at RT for at least 30 minutes and subsequently incubated at 37°C for 10 minutes. All tests were performed at 37°C. Blood was diluted 1:4 in HBS. From the diluted blood, 5 μl was added to the tubes with reaction mixture (20 μl, final dilution 1:20). In addition, 5 μl of anti-mouse Ig κ particles was added to the PC tube. Tests were incubated for exactly 20 minutes at 37°C. Reactions were stopped by adding 250 μl fixation solution (137 mmol/L NaCl, 2.7 mmol/L KCl, 1.12 mmol/L NaH_2_PO_4_, 1.15 mmol/L KH_2_PO_4_, 10.2 mmol/L Na_2_HPO_4_, 4 mmol/L EDTA, 0.5% formaldehyde). Samples were analysed 1 day after the experiment. Flow cytometry was used to discriminate platelets from other cells, using the forward and side scatter pattern and by gating on the CD41a positive cells. Median fluorescent intensity in the FITC gate was selected to determine VWF-platelet binding. Results are corrected for the MFI in the unstimulated test condition and expressed as a % of the MFI for the PC beads.

**References**

1. Hulstein JJ, de Groot PG, Silence K, Veyradier A, Fijnheer R, Lenting PJ. A novel nanobody that detects the gain-of-function phenotype of von Willebrand factor in ADAMTS13 deficiency and von Willebrand disease type 2B. Blood. 2005;106(9):3035-42.
